# Fatty acid synthase inhibitor cerulenin hinders liver cancer stem cell properties through FASN/APP axis as novel therapeutic strategies

**DOI:** 10.1016/j.jlr.2024.100660

**Published:** 2024-09-26

**Authors:** Liang-Yun Chen, Dao-Sian Wu, Yao-An Shen

**Affiliations:** 1 Department of Pathology, School of Medicine, College of Medicine, Taipei Medical University, Taipei, Taiwan; 2 Graduate Institute of Clinical Medicine, College of Medicine, Taipei Medical University, Taipei, Taiwan; 3 International Master/Ph.D. Program in Medicine, College of Medicine, Taipei Medical University, Taipei, Taiwan

**Keywords:** cancer stem cell, liver cancer, fatty acid metabolism, combination therapy, amyloid precursor protein

## Abstract

Hepatocellular carcinoma (HCC) poses significant treatment challenges due to high postoperative recurrence rates and the limited effectiveness of targeted medications. Researchers have identified the unique metabolic profiles of cancer stem cells (CSCs) as the primary drivers of cancer recurrence, metastasis, and drug resistance. Therefore, to address the therapeutic conundrum, this study focused on rewinding metabolic reprogramming of CSCs as a novel therapeutic strategy. HCC CSCs exhibited elevated fatty acid (FA) metabolism compared with parental cells. To specifically target FA metabolism in CSCs, we utilized cerulenin, a fatty acid synthase (FASN) inhibitor. Surprisingly, cerulenin can diminish CSC-like characteristics, including stemness gene expression, spherogenicity, tumorigenicity, and metastatic potential. In addition, sorafenib, a multikinase inhibitor used as targeted therapy for advanced HCC, was employed in combination with cerulenin, demonstrating a great synergistic effect, particularly in CSCs. Importantly, our RNA sequencing analysis disclosed that the amyloid protein precursor (APP) is a crucial downstream effector of FASN in regulating CSC properties. We found that APP plays a crucial role in CSCs’ characteristics that can be inhibited by cerulenin. By focusing on FA metabolism, this study identified the FASN/APP axis as a viable target to develop a more potent therapy strategy for advanced HCC.

## Graphical abstract

**Figure:**
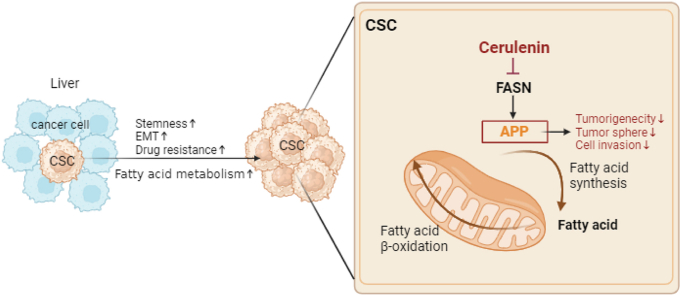

Liver cancer, particularly hepatocellular carcinoma (HCC), ranks as the sixth most commonly diagnosed cancer and the third leading cause of cancer-related death worldwide (1, 2). Metabolic diseases with risk factors such as obesity, type 2 diabetes, non-alcoholic fatty liver disease, and non-alcoholic steatosis-alcoholic steatohepatitis have been identified as potential etiologies of HCC. In Western and industrialized countries, alcohol and non-alcoholic fatty liver disease have surpassed viral hepatitis as the main cause of cirrhosis (3, 4), highlighting the significance of lipid accumulation in cancer development. Treatments for relatively early-stage HCC include radiofrequency ablation, resection, transplantation, percutaneous ethanol injections, or transcatheter arterial chemoembolization (5). Surgery is often the ultimate strategy for treatment, and targeted therapy becomes appropriate for patients with unresectable advanced HCC due to the ineffectiveness of chemotherapy drugs (6). However, the most challenging aspect of treating liver cancer is its difficulty in being detected at an early stage and its propensity for metastasis. Additionally, the postoperative recurrence rates are relatively high, jeopardizing overall patient survival (2). Sorafenib and lenvatinib are oral multiple kinase inhibitors currently used as first-line molecularly targeted drugs for advanced stages of HCC (7, 8). However, patients initially responding to these targeted drugs often develop drug resistance afterwards (8). Recent accumulating studies have explored additional combination therapy strategies to surmount resistance to sorafenib and lenvatinib (8), and the potential targets have been revealed (9, 10).

Cancer stem cells (CSCs) are an exclusive subpopulation of cancer cells that are responsible for malignant tumor development such as tumorigenesis, chemoresistance, radioresistance, and tumor relapse in different types of cancer (11, 12, 13). Importantly, CSCs possess heterogenic metabolic phenotypes and utilize different energy resources compared to general cancer cells, contributing to their survival advantage. Unlike regular cancer cells that primarily derive energy through glycolysis in the microenvironment, CSCs demonstrate metabolic plasticity by adopting preferred metabolic pathways to meet the high energy demands for tumor proliferation (14). Studies have indicated that CSCs present several main metabolic traits in different tumors, including oxidative phosphorylation (OXPHOS), glycolysis, glutaminolysis, and fatty acid (FA) metabolism (15).

Recent evidence has illustrated the significant role of FA metabolism in cancer, particularly in CSCs, emphasizing the importance of lipids or FA as essential energy resources in tumor progression (8, 9, 16). Notably, stearoyl-CoA desaturase 1 (SCD1) has been identified as a vital regulator of CSC maintenance in HCC, and its inhibition, along with the PPARα pathway, leads to the loss of CSC properties by impeding Wnt/β-Catenin signaling (17). Additionally, the examination of lipid metabolism has revealed the accumulation of neutral lipids in HCC CSCs following long-term treatment with sorafenib, accompanied by increased expression of the lipogenic transcription factor SREBP1c and enzymes involved in de novo lipid synthesis, including fatty acid synthase (FASN), ATP-citrate lyase (ACLY), and acetyl-CoA carboxylase (ACC) (18). These findings suggest that targeting the lipid metabolism of liver CSCs may be a promising strategy to counteract chemotherapy resistance. It is widely recognized that the aberrant activation of FASN may represent a significant metabolic event during the progression of HCC (8, 19, 20). Despite this, there have been limited studies investigating the beneficial effects of FASN and its underlying mechanisms in liver CSCs.

In this study, we enriched the CSC subpopulation, and the diverse metabolic biomarkers and features between parental cells and CSCs were dissected. This study also explored the synergistic effect of sorafenib and cerulenin, aiming to develop a more effective treatment strategy. We also investigated the malignancy of CSCs following the inhibition of FASN by cerulenin and analyzed the potential downstream pathways and biomarkers. Amyloid protein precursor (APP) aggregation was previously thought to be involved in the advancement of Alzheimer’s disease, and alterations in the expression of the *APP* gene may impact the disease’s susceptibility (21). Importantly, this study discovered that APP may be a critical downstream target following FASN inhibition. Ultimately, we aimed to thoroughly explore the underlying mechanisms following FASN/APP modulation in the future, providing innovative therapeutic strategies for eradicating liver CSCs.

## Materials and Methods

### Cell culture

HepG2/C3A cells were cultured in Minimum Essential Medium (MEM) (Cat. MT-10-010-CV, Corning) supplemented with 10% fetal bovine serum (FBS) (Cat. 10437028, Gibco™ Thermo Fisher Scientific), and Huh7 was cultured in Dulbecco’s modified Eagle’s medium (DMEM) (Cat. 11965092, Gibco™ Thermo Fisher Scientific) containing 10% FBS, and both were supplemented with 1% PSA antibiotic solution (penicillin, streptomycin, and amphotericin B). We established liver CSC models using our previous method (22). The cell lines were incubated at 37°C in a humidified atmosphere with 5% CO_2_.

### Quantitative real-time PCR

RNA extraction was performed using the TriRNA Pure Kit (GZX050/100/200, Geneaid), and cDNA synthesis was performed using the SensiFAST™ cDNA Synthesis Kit (Cat. BIO-65054, Bioline) with 1 μg of RNA. Quantitative real-time PCR (qPCR) was performed with the SensiFAST™ SYBR Hi-ROX Kit (BIO-92005, Bioline). Primers were used at a concentration of 0.4 μM in a final reaction volume of 10 μl. The EEF1A1 expression was used as an internal control. PCR conditions were as follows: 2 min at 95°C, followed by 40 cycles of 95°C for 5s, 60°C for 10s, and 72°C for 15s. Primer sequences are detailed in Table 1.

**Table 1:** Key primers used in the present study

Gene symbol	Sequence 5′-3′
OCT4	F: CTGGGTTGATCCTCGGACCT
	R: CACAGAACTCATACGOCGGG
SOX2	F. TACAGCATGTCCTACTOGCAG
	R: GAGGAAGAGTACCACAGGG
KLF4	F: CGGACATCAACGACGTGAG
	R: GACGCCTTCAGCACGAACT
NANOG	F: TTTGTGGGCCTGAAGAAAACT
	R: AGGGGTGTCCTGAATAAGCAG
ABCG2	F: ACGAACGGATTAACAGGGTCA
	R: CTCCAGACACACCACOGAT
MDR-1	F: GGGATGGTCAGTGTTGATGGA
	R: GCTATOOTGOTGGCAAACAATA
E-CADHERIN	F: TOCOCAGAATGAAAGG
	R: GTGTATGTOGCAATGCGTTC
VIMENTIN	F. GAGAACTTTGCCGTTGAAGC
	R GCTTCCTGTAGGTGGCAATC
TWIST	F:GGAGTCCGCAGTCTTACGAG
	R: TCTGGAGGACCTGGTAGAGG
SNAIL	F: CCTCCCTOTCAGATGAGGAC
	R: CCAGGCTGAGGTATTCCTTG
SLUG	F: GGGGAGAAGCCT TTTTCTTO
	R: TCCTCATGTTTGTGCAGGAG
ZEB1	F: ACTOCTGGGAGGATGACACA
	R: ATOCTOCTTCATOTOCCTGA
SCD1	F: GCCCCTCTACTTGGAAGACGA
	R: AAGTGATCCCATACAGGOCTC
FABN	F: ACAGCGGGGAATGGGTACT
	R: GACTGGTACAACGAGCGGAT
SREBP1	F: CGGAACCATCTTGGCAACAGT
	R: COCTTCTCAATGGCGTTGT
ACC	F: ATGTCTGGCTTOCACCTAGTA
	R: CCCCAAAGOGAGTAACAAATTCT
ACLY	F: ATOOGTTCAAGTATGCTCGGG
	R. GACCAAGTTTTCCACGACGTT
HK2	F: CCCTGCCACCAGACТАААСТ
	R: TGGACTTGAATCCCTTGGTC
GPI	F: TCTATGCTCCCTCTGTGTTAGA
	R: CTCCTCCGTGGCATCTTTATT
PFKM	F: AGGAGGGAAGGGCATCT
	R: TTCCTATCAAATGGGGTTGG
GADPH	F: GGTGTGAACCATGAGAAGTATGA
	R: GAGTCCTTCCACGATACCAAAG
LDHA	F: AGATTCCAGTGTGCCTGTATG
	R: ACCTCTTTCCACTGTTCCTTATC
PDK	F: ACGCTGGGTAATGAGGATTTG
	R: GAGGTCTTGGTGCAGTTGAATA
PDHA1	F: AGGTTTGCTGCTGCCТАТТ
	R: ACTCCAGGGTCACTCATACT
EEF1A1	F: GCTGGAAGATGGCCSTAAAAT
	R: CCAAAGGTGGATAGTCTGAGAAG
APP	F: CAGTCTGCCACAGAACATGG
	R: GGTTGGCACTGCTCCTG
MAPK8	F: CTGGCTGGAATCTAGCAGTCT
	R: CATGTCTGAAGCGCAGTAAGATT
AKT1	F: TCCTCCTCAAGAATGATGGCA
	R: GTGCGTTCGATGACAGTGGT
EGF	F: TGTCCACGCAATGTGTCTGAA
	R: CATTATCGGGTGAGGAACAACC

### Western blotting

Cells were harvested by scraping into cell lysis buffer (Cat. 9803S, Cell Lysis Buffer (10×), Cell Signaling Technology) on ice for 2 h. Lysates were centrifuged at 20,000 g for 10–20 min, and the supernatant was transferred to the new tubes. The measurement of the protein concentration was done by using a BCA assay in a 96-well plate. Samples were mixed with 6× sample dye and were next denatured at 95°C for 10 min 30–50 μg of total protein was then loaded onto sodium dodecyl sulfate-polyacrylamide gels (SDS-PAGE) and run at 90–150V. Proteins were transferred to the PVDF membrane via the Trans-Blot Turbo Transfer System from Bio-Rad. Membranes were blocked in 5% BSA in 0.01% TBST for 1 h at room temperature and incubated with primary antibodies, which were used at concentrations recommended by the manufacturer, overnight on the shaker at 4°C. The next day, after the membranes were washed three times for 10 min in 0.1% TBST, second antibodies at a concentration of 1:5000 were added. Membranes were incubated with antibodies for 1 h on the shaker at room temperature and then washed three times likewise. After being covered with the prepared HRP chemiluminescent substrate, proteins could be detected by emitting light.

### Seahorse XFe analysis

Cells were seeded in the Seahorse XFe 24-well microplate (Cat. 102,340-100, XFe24 FluxPaks, Agilent) in MEM (HepG2/C3A cells) or DMEM (Huh7 cells), and the Seahorse XFe sensor probes were activated via adding 1000 μl Calibrant Solution (Cat. 100,840-000, Agilent) in a non-CO_2_ incubator at 37°C overnight. After incubation overnight, the medium was changed to assay medium (Cat. 103575-100, DMEM, Agilent) with 1% 2 mM L-glutamine. The Seahorse XFe Mitostress Test was then run using the 3-injection protocol. The working concentration includes 1 μM oligomycin, 1 μM FCCP, and 0.5 μM rotenone/antimycin A.

### Cell viability test

Cells at a density of 1 × 10^4^ cells per well were seeded in 96-well plates overnight. Sorafenib was used at a working concentration of 10 μM with sequencing 3 times dilution to 0.12 μM, while cerulenin was used at 250 μM with sequencing 3 times dilution to 1.03 μM. Cells were treated with cerulenin (Item No. 10005647, Cerulenin, Cayman Chemical) and sorafenib prospectively or in combination for 48-h culture. Cell viability was observed by the PrestoBlue™ Cell Viability Reagent, and cells were counted using the SpectraMax M2e Microplate Reader (SpectraMax® M2e Microplate Reader, Molecular Devices LLC).

### Tumor sphere formation assay

The HepG2/C3A and Huh7 CSCs (5 × 10^4^/well) were seeded and treated in 6-well plates, which were pre-coated with 1 ml of 1.2% agarose and filled with the 5% FBS medium for sphere culture. After 72 h of incubation, the cells were photographed, and the spheres that reached a diameter of 100 μm were calculated.

### Soft agar assay

A total of 5 × 10^4^ cells were suspended in 0.4% low-melt agarose and then seeded onto the 6-well plates, which were precoated with 1 ml of 1.2% agarose. 2 ml of the 5% FBS-containing medium were added on top of the agarose layer. After 5–7 days of incubation, the colonies with more than 20 cells were counted.

### Transwell invasion assay

The 100 μl of HepG2/C3A and Huh7 CSCs (10^4^/well) were seeded and treated onto the upper compartment of the transwell chambers, which was coated with a mixture of serum-free medium and matrigel 2:1, and the lower compartment was filled with 600 μl of 10% FBS medium. After incubation for a week, the invaded cells on the upper surface of the chamber were stained by crystal violet. Three fields were photographed in each well.

### Cell migration assay

The inserts (Cat. 80209, ibidi GmbH) were first adhered to the 24-well plate. The 70 μl of HepG2/C3A and Huh7 CSCs (2 × 10^4^/well) were seeded on both sides of the insert. Once the cells adhered to the plate, we removed the insert and created a gap between the cells. The gap healing condition would be photographed every day to investigate the cell migration capability.

### RNA sequencing

The RNA samples of cell lines, including Huh7 CSCs and cerulenin-treated Huh7 CSCs, were collected. RNA purity and quantification were evaluated by SimpliNano™ Biochrom Spectrophotometers (Biochrom). The Qsep 100 DNA/RNA Analyzer (BiOptic Inc) was used to monitor the degradation and integrity of RNA. 1 μg of total RNA per sample was utilized for the RNA sample preparation. The mRNA was extracted from total RNA with magnetic oligo-dT beads. The isolated mRNA was then fragmented in the presence of magnesium in KAPA Fragment, Prime, and Elute Buffer (1×). The first strand of cDNA was synthesized through random hexamer priming. The combined second-strand synthesis and A-tailing process incorporated dUTP into the second cDNA strand and added dAMP to the 3′ ends of the resulting double-stranded cDNA (dscDNA). The dsDNA adapter with 3′dTMP overhangs was then ligated to library insert fragments. cDNA fragments of around 300–400 bp in length were preferentially selected, and the library fragments were purified using the KAPA Pure Beads system (KAPA Biosystems). The library underwent amplification using KAPA HiFi HotStart ReadyMix (KAPA Biosystems). The strand labeled with dUTP was not amplified, enabling strand-specific sequencing. Subsequently, PCR products were purified using the KAPA Pure Beads system. The library’s quality was assessed with the Qsep 100 DNA/RNA Analyzer (BiOptic Inc), the Qubit® 2.0 Fluorometer (Thermo Scientific), and the Agilent Bioanalyzer 2100 system. The library was finally sequenced through an Illumina NovaSeq6000 platform, resulting in the generation of 150-bp paired-end reads.

### Data analysis of RNA sequencing

The initial sequence data generated by the high-throughput sequencing (Illumina NovaSeq 6000 platform) underwent initial processing through CASAVA base calling, resulting in raw sequenced reads in FASTQ format. Quality assessment was conducted using FastQC and MultiQC (23) on the fastq files. Subsequently, Trimmomatic (24) was utilized to eliminate low-quality reads and trim adapter sequences, resulting in high-quality data for downstream analysis. The read pairs from each sample were then mapped to the corresponding reference genome using HISAT2 (25, 26), and featureCounts (27) could quantify the number of reads mapped to individual genes. Two types of normalization were executed, depending on the presence of biological replicates. For experiments lacking biological replicates, the “Trimmed Mean of M-values” (TMM) normalization was implemented using edgeR (28). On the other hand, experiments with biological replicates underwent "Relative Log Expression" (RLE) normalization with the involvement of DESeq2 (29, 30). For the detection of differentially expressed genes (DEGs) under two different conditions, we utilized DEGseq (for experiments lacking biological replicates) and DESeq2 (for experiments with biological replicates) (31, 32, 33, 34). An array of enrichment analyses and network analyses were performed to gain further understanding of the DEGs. Initially, the *P*-values obtained from DEG detection were adjusted through Benjamini and Hochberg's approach. Furthermore, Gene Ontology (GO) and KEGG pathway (35, 36) enrichment analyses of DEGs were conducted using clusterProfiler (37), while Disease Ontology (DO) enrichment analysis was carried out employing the DOSE package against DO, DisGeNET and NCG databases (38, 39, 40, 41). In addition, we employed Gene Set Enrichment Analysis (GSEA) (42) to discover enriched biological functions and activated pathways from the molecular signatures database (MSigDB) with 1,000 permutations. (43). Following this, we developed a protein-protein interaction (PPI) network for DEGs using STRINGdb. Subsequently, Weighted Gene Co-expression Network Analysis (WGCNA) constructed a co-expression network and executed it with the WGCNA package in R (44, 45). We employed rMATS to identify alternative splicing events corresponding to five major types of alternative splicing patterns. (46). By analyzing the isoform ratio of a gene between two conditions with rMATS, we calculated the *P*-value and false discovery rate of the difference.

### Lentiviral infection and gene knockdown

After the extraction of lentiviral vectors, the vectors were packaged by pCMV-ΔR8.91 and pMD.G plasmids. The mixture of three plasmids was transfected into 293T cells to generate larger quantities of useable lentivirus, which were harvested 48 and 72 h after transfection and then purified. Then, to infect CSCs with the collected virus, both HepG2/C3A and Huh7 CSCs were seeded in 6 cm plates and infected with APP shRNA lentivirus (TRCN0000011043, TRCN0000006705, TRCN0000006707, TRCN0000011042, shown as shAPP#1, shAPP#2, shAPP#3, and shAPP#4, RNA Technology Platform and Gene Manipulation Core), which was previously collected. After 24 h of culture, the virus and polybrene (8 μg/ml) were added to the fresh medium. We have used puromycin (2–5 μg/ml) for selection. For further investigation the correlation between APP and cerulenin, the APP expression would be rescued by recombinant APP (Cat. 787606, BioLegend).

## Results

### Liver CSCs exhibited increased expression of stemness and altered metabolic signatures

**Fig. 1:**
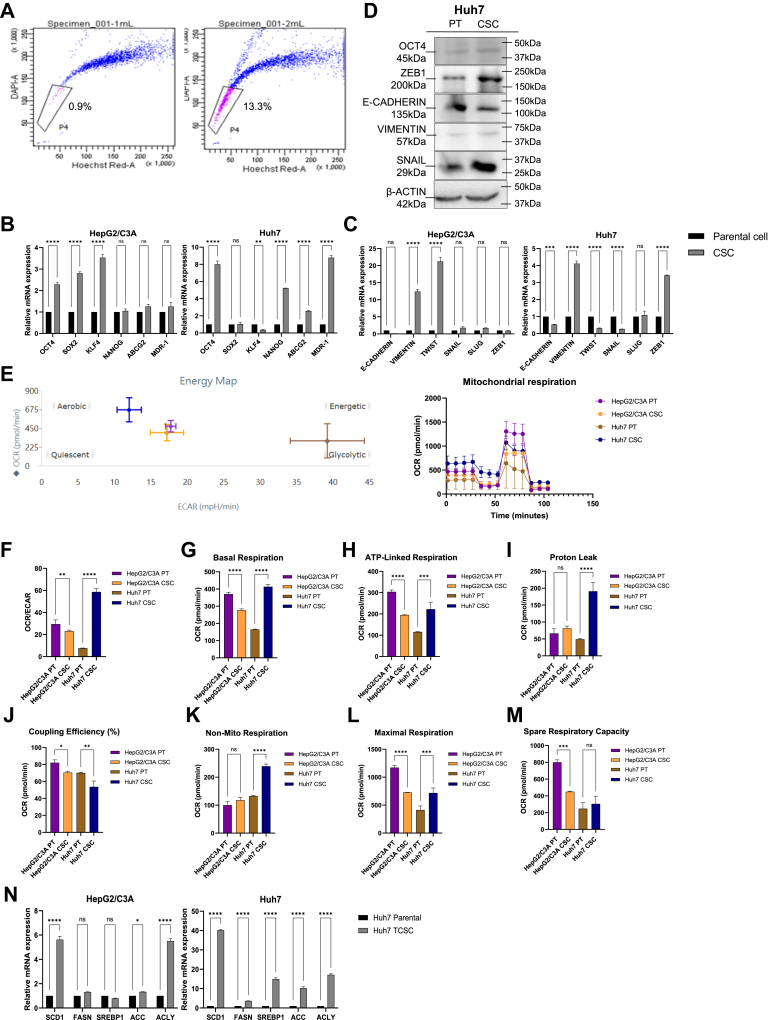
CSC-like features enhanced and metabolism reprogrammed in the liver CSC model. A: Side population selection of the liver CSC model. B: The mRNA levels of the representative markers of CSC-like characteristics, including stemness and drug resistance, in two distinct CSC models were measured by qPCR. ∗∗, *P* < 0.01; ∗∗∗∗, *P* < 0.0001; ns, not statistically significant; Student *t* test. C: The mRNA levels of the representative markers of EMT in two distinct CSC models were measured by qPCR. ∗∗∗, *P* < 0.001; ∗∗∗∗, *P* < 0.0001; ns, not statistically significant; Student *t* test. D: Western blotting of the markers of CSC-like characteristics in the CSC model. E: Seahorse XFe analysis of the metabolic tendency in the parental cells and CSCs under 1 μM FCCP treatment. Values for the following respiratory parameters were determined. F: OCR/ECAR. ∗∗, *P* < 0.01; ∗∗∗∗, *P* < 0.0001; Student *t* test. G: Basal respiration. ∗∗∗∗, *P* < 0.0001; Student *t* test. H: ATP-linked respiration. ∗∗∗, *P* < 0.001; ∗∗∗∗, *P* < 0.0001; Student *t* test. I: Proton leak. ∗∗∗∗, *P* < 0.0001; ns, not statistically significant; Student *t* test. J: Coupling efficiency (%). ∗, *P* < 0.05; ∗∗, *P* < 0.01; Student *t* test. K: Non-mitochondrial respiration. ∗∗∗∗, *P* < 0.0001; ns, not statistically significant; Student *t* test. L: Maximal respiration. ∗∗∗, *P* < 0.001; ∗∗∗∗, *P* < 0.0001; Student *t* test. M: Spare respiratory capacity. ∗∗∗, *P* < 0.001; ns, not statistically significant; Student *t* test. N: The mRNA levels of the representative markers of FA metabolism in two distinct CSC models were measured by qPCR. ∗, *P* < 0.05; ∗∗∗∗, *P* < 0.0001; ns, not statistically significant; Student *t* test.

To identify the potential metabolic target of CSCs, we established stable liver CSCs by behavior selection, as described previously (22). After sorting out 13.3% of CSCs by side population selection (Fig. 1A), we analyzed their stemness genes, including *OCT4*, *NANOG*, *SOX2*, *KLF4* (Fig. 1B), chemoresistant genes like *ABCG2* and *MDR-1* (Fig. 1B), and epithelial-mesenchymal transition (EMT)-related genes like *E-CADHERIN*, *VIMENTIN*, *TWIST*, *SNAIL*, and *SLUG* (Fig. 1C). While HepG2/C3A and Huh7 CSCs generally manifested upregulated stemness and EMT gene expressions compared to their corresponding parental cell lines, certain genes displayed differential expression patterns. We also validated the presence of CSC-associated markers in these two cell lines through Western blot analysis (Fig. 1D).

Metabolic reprogramming is critical for energy acquisition and survival in CSCs. This includes the metabolic shift to predominately rely on fatty acids, mitochondria, glycolysis (47, 48), and redox metabolism (49). To further assess their metabolic functions, we use the Seahorse XFe analysis to observe the altered metabolic patterns in CSCs. We evaluated the oxygen consumption rate (OCR) and extracellular acidification (ECAR) before and after the addition of inhibitors and compared the mitochondrial respiration of parental cells and CSCs (Fig. 1E). Intriguingly, HepG2/C3A and Huh7 CSCs exhibited differential metabolic shifts (Fig. 1E). In the energy map, Huh7 CSCs shift dramatically to a more aerobic status, whereas HepG2/C3A CSCs shift slightly to a quiescent status (Fig. 1E). The OCR/ECAR ratio, basal respiration, and ATP synthase activity all showed the same trend in Huh7 CSCs. This showed that the cells’ metabolism changed from glycolysis to OXPHOS for energy production, which resulted in much higher mitochondrial ATP production (Figs. 1F–H). However, the backflow of protons resulted in an impedance to coupling efficiency (50). We found that there was a higher trend in proton leak in Huh7 CSCs (Fig. 1I), which led to lower coupling efficiency (though it was not statistically significant) (Fig. 1J). We observed an increase in non-mitochondrial respiration in CSCs compared to parental cells (Fig. 1K). To withstand stress, such as oxidative stress, Huh7 CSCs showed stronger maximal and spare respiratory capacity (Fig. 1L, M). HepG2/C3A CSCs, on the other hand, had lower OCR/ECAR ratio (Fig. 1F), basal respiration (Fig. 1G), ATP synthase activity (Fig. 1H), coupling efficiency (Fig. 1J), maximal (Fig. 1L), and spare (Fig. 1M) respiratory capacity compared with parental cells. To further examine their metabolic regulations, we analyzed the levels of gene expression associated with fatty acid metabolism, including *SCD1*, *FASN*, *SREBP1*, *ACC*, and *ACLY,* in these liver CSCs. The qPCR results demonstrated a significantly elevated expression of FA metabolism in CSCs (Fig. 1N). Conclusively, this investigation demonstrated that CSCs may adjust their energy sources from FA in order to achieve metabolic reprogramming and metabolic plasticity.

### FASN inhibition reduced the tumorigenicity and spherogenesis of liver CSCs

**Fig. 2:**
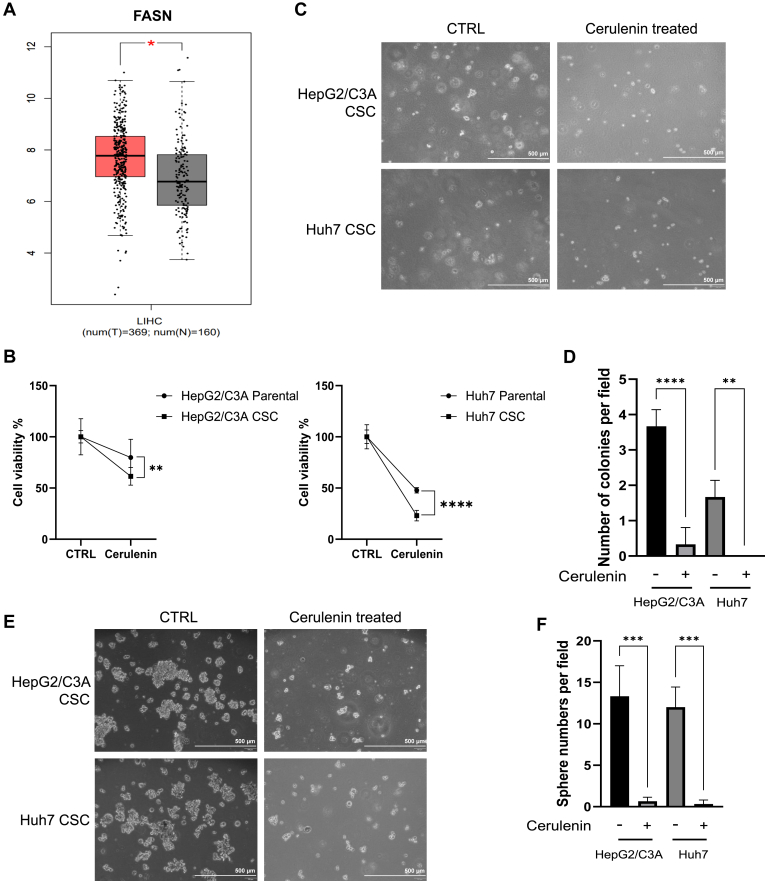
Cerulenin treatment impacted tumorigenic capacity in liver CSCs in vitro. A: expression of FASN in HCC tissues (n = 369) and normal liver tissues (n = 160) from the TCGA and GTEx databases LIHC: liver hepatocellular carcinoma. B: cell viability test of the parental cells and CSCs following deprivation of essential nutrients. CTRL: The cells were not treated. Cerulenin: The cells were treated with cerulenin at concentrations of IC50. ∗∗, *P* < 0.01; ∗∗∗∗, *P* < 0.0001; Student *t* test. C: soft agar assay. Cerulenin treated: The cells received a 20 μM concentration of cerulenin treatment (n = 3). D: the quantitative results of the cells from the soft agar assay. ∗∗, *P* < 0.01; ∗∗∗∗, *P* < 0.0001; Student *t* test. E: Tumor sphere formation assay. Cerulenin treated: The cells were treated with cerulenin at a concentration of 20 μM (n = 3). F: The quantitative results of the cells from the tumor sphere formation assay. ∗∗∗, *P* < 0.001; Student *t* test.

Cerulenin is a specific FASN inhibitor possessing the capability to suppress malignant phenotypes and induce apoptosis in cancer cells (51, 52). Researchers have demonstrated the inhibitory effect of cerulenin on liver cancer cells (53). However, the impact and the inhibitory mechanisms in CSCs remain ambiguous (54, 55). Therefore, we surveyed the cerulenin-mediated underlying mechanisms in liver CSCs. Given that the qPCR results demonstrated a significant increase in FA metabolism involvement in CSCs and supporting data from the TCGA and GTEx databases also indicated a significant difference in the expression of FASN in HCC tissues and normal liver tissues (Fig. 2A), suggesting the crucial role of FASN in cancer development, we further selected FASN as the target and hypothesized that inhibiting FASN could impact CSC survival. For that glucose and glutamine catabolism serve as crucial nutrients for cancer cell survival (56), we cultured parental cells and CSCs in a medium devoid of glucose and glutamine to assess the specific inhibitory effect of cerulenin on survival, thereby preventing potential compensatory effects from alternative metabolic pathways. The working concentration of cerulenin was used at IC50 in HepG2/C3A and Huh7, respectively (Supplemental Fig. S1). The cell viability test also demonstrated that CSCs were more vulnerable to low doses of cerulenin but were more resistant to high doses of cerulenin (Supplemental Fig. S1). Here, with the culture medium devoid of glucose and glutamine, we found that cerulenin had a considerable inhibitory effect on liver cancer cells, with CSCs showing greater sensitivity to cerulenin compared to parental cells (Fig. 2B). The soft agar assay (Fig. 2C) demonstrated a reduction in tumorigenicity in cerulenin-treated CSCs in comparison to non-treated CSCs, particularly in Huh7, with a statistically significant difference (Fig. 2D). In the tumor sphere-forming assay (Fig. 2E), the size and number of the tumor spheres significantly declined in HepG2/C3A and Huh7 CSCs, implying cerulenin can significantly suppress long-term self-renewal and anti-anoikis capacity (Fig. 2F). In conclusion, the metabolism of fatty acids played a vital role in maintaining the survival and ability to form tumors and spheroids in CSCs.

### FASN inhibition suppressed the metastatic potential of liver CSCs

**Fig. 3:**
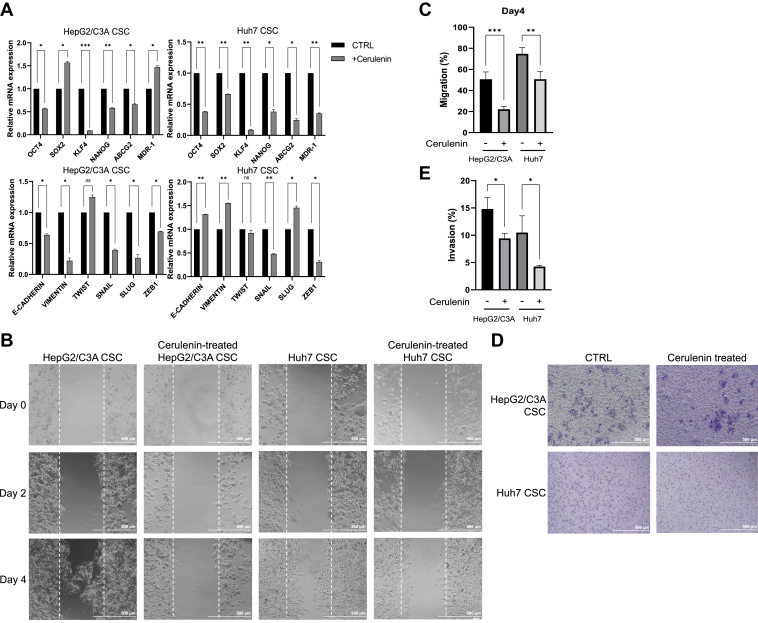
Migration and invasion ability declined, and the metabolic patterns were altered in liver CSCs in vitro. A: the mRNA levels of stemness, EMT, and drug resistance-related genes in cerulenin-treated CSCs were assessed by qPCR. ∗, *P* < 0.05; ∗∗, *P* < 0.01; ∗∗∗, *P* < 0.001; ns, not statistically significant; Student *t* test. B: cell migration assay of the untreated and cerulenin-treated CSCs. Cerulenin treated: The cells were treated with cerulenin at a concentration of 20 μM. (n = 3). C: The migration ability of CSCs with and without treatment was analyzed. ∗, *P* < 0.05; ∗∗, *P* < 0.01; ∗∗∗, *P* < 0.001; Student *t* test. D: transwell invasion assay. Untreated: The cells without treatment. Cerulenin treated: The cells were treated with cerulenin at a concentration of 20 μM (n = 3). E: The invasion capacity of CSCs with and without treatment was analyzed.

Further investigation is necessary to determine whether cerulenin could impact the stemness and invasion capabilities of CSCs, in addition to the noted malignant characteristics such as tumorigenicity (57). As a result, we observed stemness features in cerulenin-treated CSCs. The qPCR results showed a notable decrease in stemness and chemoresistance genes in CSCs following cerulenin treatment, particularly in Huh7 (Fig. 3A). The alterations in EMT markers exhibited a similar trend (Fig. 3A). Cerulenin treatment attenuated the capacity for migration and invasion of CSCs in functional assays, as evidenced by cell migration and transwell invasion assays. In the cell migration assay (Fig. 3B), we observed that CSCs exhibited sluggish migration due to their tendency to develop in a layered manner rather than moving through the gap. Nevertheless, we were able to discern the distinction between the groups that were exposed to cerulenin and those that were not. Cerulenin greatly reduced the migratory capability of CSCs (Fig. 3C). Likewise, cerulenin-treated CSCs showed a reduction in their invasion ability, with HepG2/C3A CSCs demonstrating a particularly significant decrease (Fig. 3D, E). Thus, administering cerulenin can inhibit liver CSCs’ stemness features and ability to invade and migrate.

### Liver CSCs displayed enhanced synergism with cerulenin and sorafenib

**Fig. 4:**
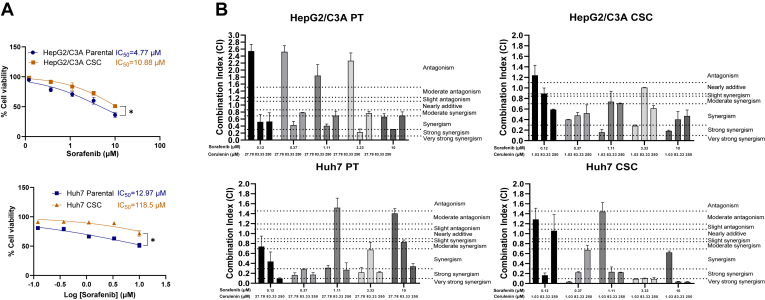
Combination therapy of sorafenib and cerulenin in liver CSCs. A: cell viability test of the parental cells and CSCs with sorafenib treatment. Sorafenib was treated at 10 μM, with sequencing three times dilution to 0.12 μM. ∗, *P* < 0.05; Student *t* test. B: the CI value of the treatment with sorafenib and cerulenin was determined. Thresholds of synergism or antagonism were noted.

Sorafenib is the current first-line targeted drug for late-stage HCC. Unfortunately, it has a limited effect on eradicating liver tumors (58, 59). To improve the cure rate of liver cancer, our objective is to investigate if cerulenin can enhance the efficacy of commonly used targeted therapy drugs. We conducted cell viability assays using HepG2/C3A and Huh7 parental cells, as well as CSCs, to assess the individual drug efficacy of sorafenib and cerulenin, along with their combination effect. The results demonstrated that both parental cells were more sensitive to sorafenib, indicating the relative resistance of CSCs to sorafenib treatment (Fig. 4A). In low-dose treatment, CSCs were more sensitive to cerulenin than parental cells, but in high-dose treatment, they were more resistant (Supplemental Fig. S1). In general, Huh7 displayed more resistance to sorafenib (Fig. 4A) and cerulenin (Supplemental Fig. S1) than HepG2/C3A.

To provide novel treatments targeting cancer metabolism for overcoming resistance, we further analyzed the synergism between sorafenib and cerulenin using CompuSyn software and assessed the combination index (CI) to identify the drug combination effects. In this assay, we combined the current liver cancer-targeted drug, sorafenib, and cerulenin with fixed concentrations as aforementioned. It is known that CI < 1 represents synergism, CI = 1 represents the additive effect and CI > 1 stands for antagonism (60). The thresholds of very strong synergism (CI < 0.1), strong synergism (CI = 0.1–0.3), synergism (CI = 0.3–0.7), moderate synergism (CI = 0.7–0.85), slight synergism (CI = 0.85–0.9), nearly additive (CI = 0.9–1.1), slight antagonism (CI = 1.1–1.2), moderate antagonism (CI = 1.2–1.45), and antagonism (CI = 1.45–3.3) were noted on the plots (61) (Fig. 4B). Our results indicated that synergism existed in HepG2/C3A and Huh7 parental cells (abbreviated as PT in the plot), especially when a high dose of cerulenin was combined (Fig. 4B). In HepG2/C3A and Huh7 CSCs, synergism was observed both in combination with low- and high-dose cerulenin. Importantly, a very strong synergism between sorafenib and cerulenin could be achieved, particularly in Huh7 CSCs (Fig. 4B). The results indicated a promising combination therapy that showed a synergistic effect on eliminating CSCs.

### APP is a critical target for regulating FA metabolism in CSCs

**Fig. 5:**
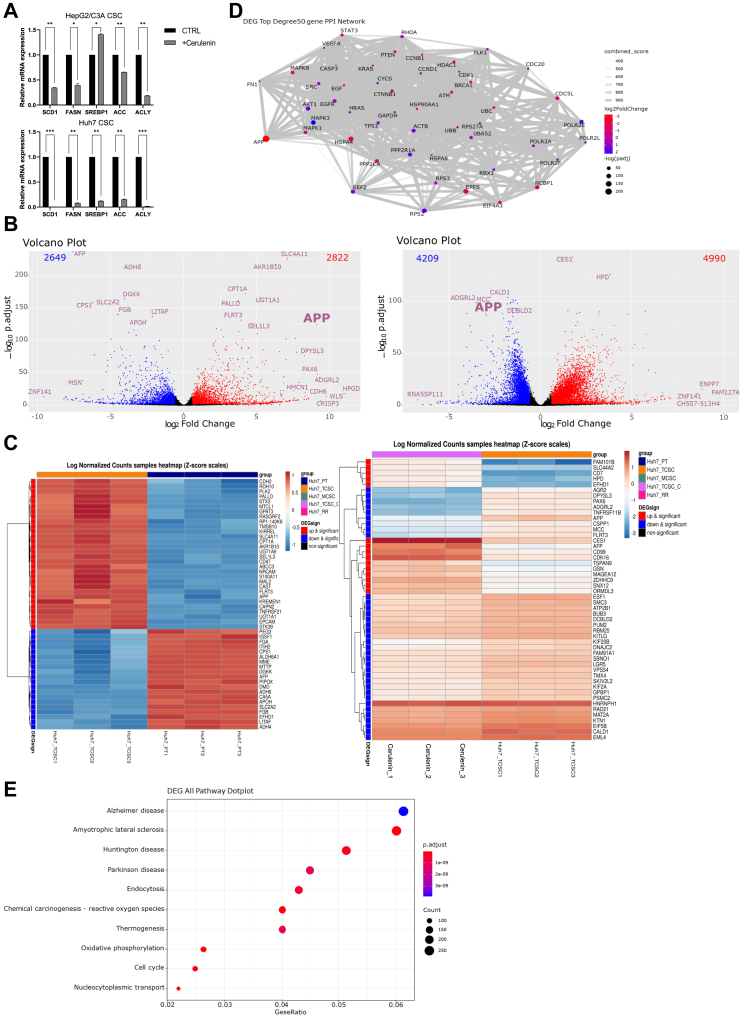
The altered metabolic patterns in liver CSCs. A: The mRNA expression of FA metabolism and glycolysis of CSCs with and without cerulenin treatment was measured by qPCR. Cerulenin was treated at a concentration of 20 μM. ∗, *P* < 0.05; ∗∗, *P* < 0.01; ∗∗∗, *P* < 0.001; Student *t* test. B: the DEG analysis was presented as volcano plots, and the APP gene was listed in both of the plots. Left: Comparison of RNA expression between CSCs and parental cells. Right: A comparison of RNA expression between cerulenin-treated and untreated CSCs. The DEG Analysis Parameter Set was established using the DEG Method. DESeq2. The *P*-value threshold was set at 0.05, and the fold change threshold was 1.5. C: RNA-sequencing sample heatmap. Left: Huh7 CSCs without treatment versus Huh7 parental cells. Right: Huh7 CSCs with cerulenin treatment at 20 μM versus Huh7 CSCs without treatment. Huh7_PT: Huh7 parental cells. Huh7_TCSC: Huh7 CSCs without treatment. Cerulenin_1/2/3: Huh7 CSCs with cerulenin treatment at a concentration of 20 μM. D: The top 50 genes, such as *APP*, according to the PPI network analysis were listed. E: DEG pathway dotplot. KEGG Analysis Parameter Set Analysis with gene set range:10–500. The adjusted *P*-value threshold was 0.05, and the fold change threshold was 1.2.

According to our research, it is now evident that the FASN inhibitor, cerulenin, has the capability to reduce CSCs’ tumorigenicity, spherogenesis, stemness features, and ability to invade and migrate. Before delving into the underlying mechanism, we confirmed the mRNA expression of biomarkers in FA metabolism. The qPCR results demonstrated that following cerulenin treatment, the expression of FASN and other FA synthesis-related markers decreased significantly, particularly in Huh7 CSCs (Fig. 5A).

In RNA sequencing analysis, the principal component analysis (PCA) visually demonstrated and presented the variance between Huh7 parental cells (referred to as Huh7_PT), CSCs with cerulenin treatment (referred to as Huh7_TCSC_C), and CSCs without cerulenin treatment (referred to as Huh7_TCSC) in 3D and 2D PCA (Supplemental Fig. S2). We discovered distinct subgroups formed by Huh7 parental cells, CSCs with cerulenin, and CSCs without cerulenin. We then investigated the significantly altered genes and pathways in CSCs compared to parental cells, as well as in CSCs with cerulenin treatment compared to CSCs without treatment. In the DEG analysis, using a threshold of adjusted *P*-value <0.05 and｜fold change｜>1.5, the volcano plot illustrated the genes that were substantially upregulated (depicted in red) and downregulated (depicted in blue), with the most significantly altered genes labeled on the plot. When comparing CSCs to parental cells, the analysis revealed 2822 upregulated gene expressions and 2649 downregulated gene expressions in CSCs (Fig. 5B). Besides, when comparing CSCs with cerulenin treatment to those without treatment, it was found that 4990 gene expressions were upregulated and 4209 gene expressions were downregulated in CSCs with cerulenin. Moreover, the heatmap displayed the top 50 altered genes (Fig. 5C). FAM101B, SLC44A2, CD7, HPD, and EFHD1 were downregulated when not treated with cerulenin but were upregulated when treated with cerulenin; AGR2, DPYSL3, PAX6, ADGRL2, TNFRSF11B, APP, CSPP1, MCC, and FLRT3 were upregulated when not treated with cerulenin but were downregulated when treated with cerulenin (Fig. 5C). This may explain why CSCs were more resistant to high-dose treatment of cerulenin compared to low-dose treatment (Supplemental Fig. S1). The cerulenin concentration in the RNA sequencing experiment was 20 μM, which was considered a low dose in the cell viability experiment setup. While HPD upregulation suggested attempts at metabolic adaptation (62), these changes might be insufficient at low doses to fully compensate for the inhibition of fatty acid synthesis. The downregulation of genes related to cell adhesion (AGR2, ADGRL2, FLRT3) (63, 64, 65) and cell cycle regulation (CSPP1, MCC) (66, 67) could potentially increase the cells’ susceptibility to cerulenin-induced stress. Changes in CD7, PAX6, and DPYSL3 expression might indicate a disruption in the balance between stemness and differentiation (68, 69, 70), potentially making the cells more susceptible to the drug’s effects at low doses. Despite the upregulation of some stress response genes (EFHD1) (70), the overall response may not be sufficiently robust at this low dose to effectively protect the cells. Downregulation of TNFRSF11B (OPG), which can act as a decoy receptor for TRAIL, might make cells more susceptible to apoptosis at low doses (71). At low doses, cerulenin induced partial metabolic stress and disrupted various cellular processes, leading to increased sensitivity. However, these changes were not severe enough to trigger a full adaptive response, and further studies are necessary to fully understand each signaling pathway and stress response.

Notably, a unique target, APP, was the only target observed in both volcano plots as well as a heatmap, signifying a significant upregulation in CSCs compared to parental cells but a downregulation after cerulenin treatment (Fig. 5B, C). Therefore, we speculated that APP may serve as a critical target, modulating CSC metabolic pathways for survival and stemness properties. In order to uncover the potential mechanism, we analyzed the protein-protein interaction (PPI) network of APP. The MAPK family appeared to play a significant role, with AKT, EGF, EGFR, FN1, and SRC being other related proteins closely related to APP (Fig. 5D). Furthermore, based on the KEGG database, Alzheimer’s disease emerged as the most prominent pathway following cerulenin inhibition, implying the potential importance of APP modulation because early-onset Alzheimer’s disease familial forms are linked to APP mutations (72) (Fig. 5E). Ultimately, the above data analyses indicated the potential pathways under cerulenin treatment, and APP seemed to be a vital downstream target.

### The APP knockdown compromised the CSC properties and the invasion potential

**Fig. 6:**
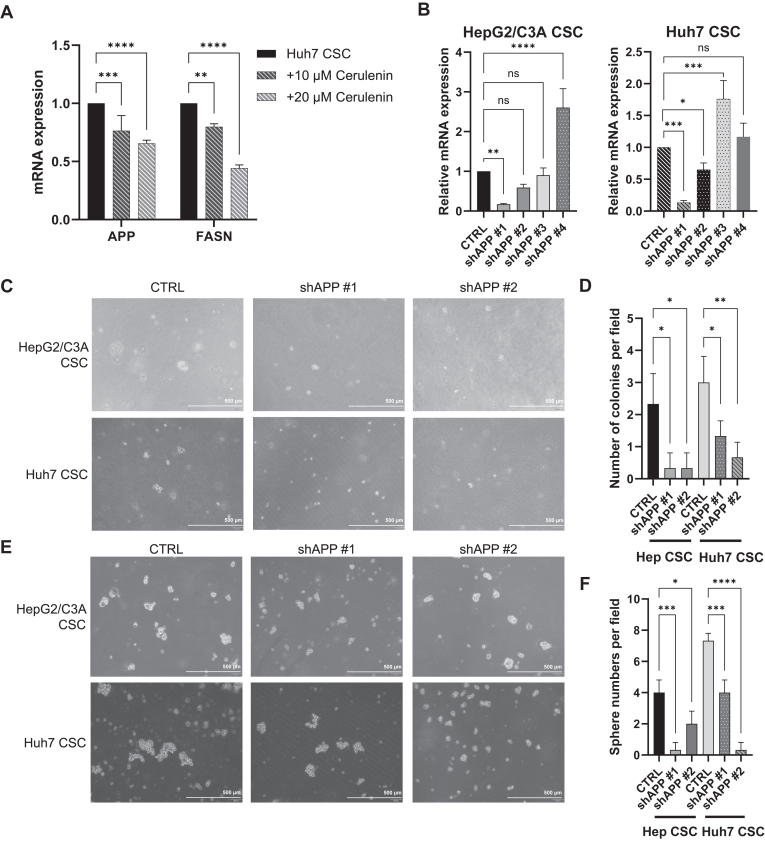
The APP/FASN modulation impacted the tumorigenicity of CSCs in vitro. (A: The mRNA expression of *APP* and *FASN* in CSCs treated with cerulenin at different concentrations (0 μM, 10 μM, and 20 μM) was measured by qPCR. ∗∗, *P* < 0.01; ∗∗∗, *P* < 0.001; ∗∗∗∗, *P* < 0.0001; two-way ANOVA. B: the qPCR results of CSCs with *APP* knocking down through four different lentiviruses, labeled as shAPP #1, shAPP #2, shAPP #3, and shAPP #4. ∗, *P* < 0.05; ∗∗, *P* < 0.01; ∗∗∗, *P* < 0.001; ∗∗∗∗, *P* < 0.0001; ns, not statistically significant; one-way ANOVA. C: soft agar assay. CTRL: The cells were not treated. shAPP #1/shAPP #2: The cells with *APP* knockdown are referred to as shAPP #1 and shAPP #2 (n = 3). D: cell colonies with more than 20 cells were counted. shAPP #1/shAPP #2: The cells with APP knockdown are referred to as shAPP #1 and shAPP #2. ∗, *P* < 0.05; ∗∗, *P* < 0.01; one-way ANOVA. E: tumor sphere formation assay. CTRL: The cells were not treated. shAPP #1/shAPP #2: the cells with *APP* knocking down (n = 3). F: spheres with at least a diameter of 100 μm were calculated. ∗, *P* < 0.05; ∗∗∗, *P* < 0.001; ∗∗∗∗, *P* < 0.0001; one-way ANOVA.

APP dysregulation is associated with various cancer processes, including cell proliferation, migration, invasion, and chemoresistance (73, 74, 75). Nevertheless, it remains unknown whether APP could directly have an impact on CSC survival and progression. In addition, the association between APP and FASN requires further research. To investigate whether it affects APP, we inhibited FASN using various concentrations of cerulenin. By using cerulenin at 0, 10, and 20 μM, we observed a dose-dependent inhibitory effect on APP (Fig. 6A). Different amounts of cerulenin treatment revealed a positive correlation between FASN regulation and APP (Fig. 6A). We used lentiviral-based shRNA to knock down *APP* gene expression in the CSC model to better understand the underlying mechanisms modulating APP. Using qPCR assays, we selected shAPP#1 and shAPP#2, two lentiviruses with significant APP knockdown efficiency, for further experiments (Fig. 6B).

**Fig. 7:**
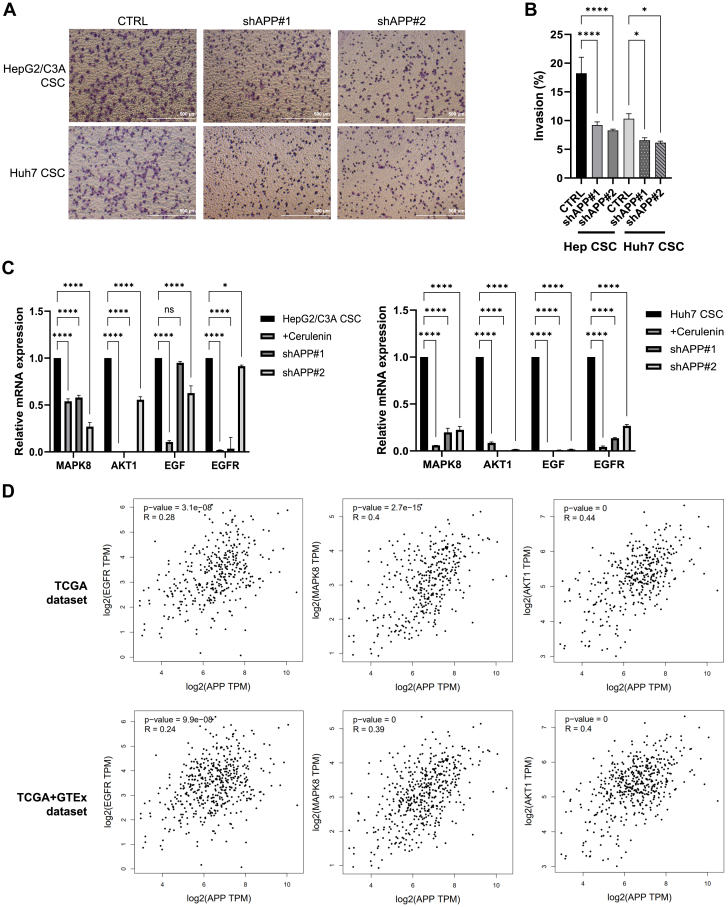
The impact of *APP* knockdown and its potential downstream pathways. A: transwell invasion assay. CTRL: The cells were not treated. shAPP #1/shAPP #2: the cells with *APP* knocking down (n = 3). B: the invasion capacity of the cells with *APP* depletion was measured. ∗, *P* < 0.05; ∗∗∗∗, *P* < 0.0001; one-way ANOVA. C: the mRNA expression of APP-related markers in CSCs treated with cerulenin 20 μM, shAPP#1, and shAPP#2 was assessed by qPCR. ∗, *P* < 0.05; ∗∗∗∗, *P* < 0.0001; ns, not statistically significant; two-way ANOVA. D: correlation analysis of APP and its downstream markers from TCGA and GTEx databases.

Subsequently, we explored the role of APP in regulating CSC functionality. The soft agar experiments show a significant decrease in colony growth in the APP-knocked groups, particularly in Huh7 CSCs (Fig. 6C, D). In tumor sphere formation assays, the sphere numbers declined after APP knockdown, and this was especially pronounced in the Huh7 cell line (Fig. 6E, F). However, in cell migration assays, APP knockdown did not show a significant effect on hindering cell migration capacity (Supplemental Fig. S3). Nevertheless, the results of transwell invasion assays indicated that APP knockdown could attenuate CSC invasion (Fig. 7A, B).

Aside from these cell functional assay findings, to further investigate potential targets involved, we measured the mRNA expression of markers shown in the previous PPI network. We found that the depletion of APP significantly suppressed the expression of MAPK8, AKT1, EGF, and EGFR in both CSC models, which also had a consistent trend under FASN inhibition (Fig. 7C). These findings revealed that FASN can regulate CSC properties and invasive capacity through the activation of APP and its downstream targets. Furthermore, correlation analysis results from the TCGA and GTE× databases also demonstrated a significant positive correlation between APP and its potential downstream markers, including EGFR, MAPK8, and AKT1 (Fig. 7D). Conclusively, knockdown of *APP* may impair tumorigenicity and invasion capacity of CSCs, and our findings indicated that MAPK8, AKT1, EGF, and EGFR may potentially be the FASN/APP downstream targets.

### APP is a crucial downstream target inhibited by cerulenin

**Fig. 8:**
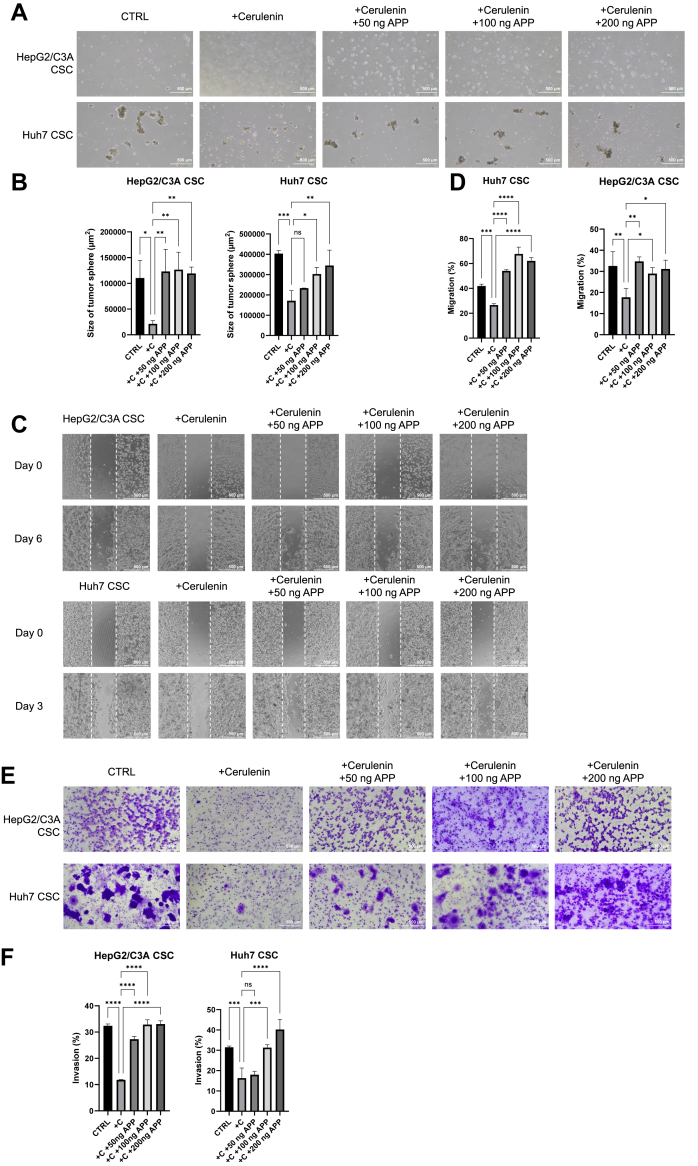
The APP rescue experiment in cerulenin-treated CSCs. A: Tumor Sphere assay. CTRL: The cells were not treated. +Cerulenin: The cells received a 20 μM concentration of cerulenin treatment (n = 3). +50, 100, 200 ng APP: The cells received a 50, 100, 200 ng of APP treatment (n = 3). B: Tumorigenicity of the cells treated with cerulenin was rescued when additional APP was added. ∗, *P* < 0.05; ∗∗, *P* < 0.01; ∗∗∗, *P* < 0.001; ns, not statistically significant; one-way ANOVA. C: Cell migration assay. CTRL: The cells were not treated. +Cerulenin: The Huh7 CSCs received a 20 μM concentration of cerulenin treatment and the HepG2/C3A CSCs received a 10 μM concentration of cerulenin treatment (n = 3). +50, 100, 200 ng APP: The cells received a 50, 100, 200 ng of APP treatment (n = 3). D: The migration capacity of the cells treated with cerulenin was rescued when additional APP was added. ∗, *P* < 0.05; ∗∗, *P* < 0.01; ∗∗∗, *P* < 0.001; ∗∗∗∗, *P* < 0.0001; one-way ANOVA. E: Transwell invasion assay. CTRL: The cells were not treated. +Cerulenin: The cells received a 20 μM concentration of cerulenin treatment (n = 3). +50, 100, 200 ng APP: The cells received a 50, 100, 200 ng of APP treatment (n = 3). F: The invasion capacity of the cells treated with cerulenin was rescued when additional APP was added. ∗∗∗, *P* < 0.001; ∗∗∗∗, *P* < 0.0001; ns, not statistically significant; one-way ANOVA.

Given the similar trends in mRNA expression between APP-knocked and cerulenin-treated CSCs (Fig. 7C), we aim to explore how much of the effect of cerulenin is mediated through APP on CSCs. Therefore, we performed the rescue experiment that treated the CSCs with cerulenin and APP simultaneously to observe the changes in tumorigenicity, spherogenesis, and metastatic potential. The CSCs were treated with 20 μl of cerulenin and 50, 100, and 200 ng of APP. Tumor sphere assay showed rescued spherogenesis in CSCs treated with cerulenin and APP, especially a dose-dependent rescue effect in Huh7 CSCs (Fig. 8A, B). The soft agar assay showed a rescued tumorigenicity when treated with cerulenin and APP in Huh7 CSCs (Supplemental Fig. S4). We also found that the invasion and migration capacity was rescued in cell migration and transwell invasion assays (Fig. 8C–F), suggesting that APP plays a crucial role in CSCs’ characteristics that can be inhibited by cerulenin.

## Discussion

Recent studies have indicated that, in addition to consuming glucose due to the Warburg effect, cancer cells, particularly CSCs, can utilize more diverse nutrients, including FAs and amino acids, to adapt and enhance their survival (48). Lipid metabolic reprogramming is a hallmark of cancer malignancy and has received increasing interest in recent years (76). While de novo FA synthesis occurs in normal adult organisms such as adipose tissue, liver, and breast, only little endogenous FA synthesis is observed, in contrast to the high expression in several cancer cells (77). These cancer cells develop de novo FA synthesis with increased activity of multiple lipogenic enzymes, ACC, ACLY, CoA carboxylase (ACACA), and SCD (77). The overexpression and enhanced activity of FASN, a crucial metabolic multi-enzyme in modulating the terminal catalytic step in FA synthesis, leads to alterations in the phenotype of cancer cells and is often associated with a high risk of disease recurrence and death (78). Previous studies have demonstrated the role of FASN in HCC, with higher expression in liver cancer tissues compared to non-tumoral tissues (79). It has been implicated in the occurrence and metastasis of HCC (80). The β-CATENIN/C-MYC signaling pathway may be the underlying mechanism after inhibition of FASN, potentially leading to decreased proliferation and increased apoptosis in HepG2 cells (81). Furthermore, FASN appears to play a critical role in maintaining cancer stemness (82), suggesting it could be a vulnerability point of CSCs.

The EMT process has been considered to be associated with cancer malignancy and cause increases in the metastases (83). In this study, we first established a CSC model using the parental cells of HepG2/C3A and Huh7. In the EMT program, stem-like markers, including stemness and drug resistance, were consistently higher in both cell line CSCs compared to their parental cells. The further qPCR tests indicated a link between de novo synthesis of FAs and glycolysis, which provides precursors and energy for FA synthesis (78). Moreover, the Seahorse XFe analysis has analyzed the mitochondrial function of parental cells and CSCs via the real-time OCR. It was uncovered that CSCs reprogrammed their metabolism to acquire enough energy for survival by switching the tendency to mitochondrial respiration, particularly in Huh7 CSCs.

By specifying the energy source for culture, we found the cell survival dependence on FA. Similar results were presented in functional assays. We then investigated the impact of cerulenin on the stem cell-like characteristics. The results indicated that the cerulenin treatment could impede CSC progression not only at the stemness gene expressions but also in the CSC functionality. Furthermore, RNA sequencing analysis showed that cerulenin treatment significantly alters the APP in CSCs, underscoring the importance of FASN/APP in controlling CSC characteristics.

APP has a well-established role in Alzheimer’s disease, as evidenced by epidemiological studies demonstrating an inverse association between Alzheimer’s disease and cancer incidence and prevalence (84). Correspondingly, the relationship between APP and Alzheimer’s disease has been implied in our volcano plots and DEG pathway dot plots. A significant upregulation of the gene APP was highlighted in CSCs compared to parental cells, and a significant downregulation of APP in CSCs was also presented under cerulenin treatment. APP has been reported to be upregulated in breast cancer, glioblastoma, nasopharyngeal carcinoma, pancreatic cancer, colon cancer, prostate cancer, and HCC (73). Overexpression of APP in cancer cells promotes cell proliferation and migration, reduces apoptosis, decreases treatment sensitivity, and impairs survival rates (73), which matches our rescue experiment. By providing additional APP to cerulenin-treated CSCs, their tumorigenicity, spherogenesis, and metastatic potential can be rescued to the level of non-cerulenin-treated CSCs. Additionally, overexpression of APP in HCC could lead to 5-fluorouracil resistance through downregulation of the mitochondrial apoptosis pathway (85). The downstream and upstream targets or signaling pathways modulated by APP in carcinogenesis could be investigated for further understanding. A product generated during APP processing, sAPPα, has been revealed to play an important role in several cancer progressions. The stimulation of EGFR by TGFα induces sAPPα secretion, leading to increased cell proliferation and motility in nasopharyngeal carcinoma (86). Similarly, the PI3K/Akt pathway is involved in the upregulation of sAPPα secretion and APP expression in thyroid cancer. Recently, the role of APP in promoting the migration and invasion of breast cancer cells through regulating the MAPK signaling pathway has also been suggested (74), which may be related to our PPI network results. However, the modulation and mechanisms between APP and FASN remain unknown. The association between the markers linked to our study, including MAPK8, AKT, EGFR, and FASN, has been investigated. It has been reported that MAPK/P53 signaling promotes bone tumor development by upregulating the expression of FASN (87). In human HCC cell lines, FASN inactivation leads to the downregulation of AKT phosphorylation, which subsequently inhibits hepatocarcinogenesis (23). In PC3 (prostate cancer) and A549 (lung cancer) cells, EGFR could be activated intracellularly via FASN-mediated protein palmitoylation, becoming a potential target for anticancer therapy (88). Our study using *APP* knockdown found that the possible downstream pathway of FASN/APP modulation may affect liver CSC tumorigenicity and invasion capacity. It was suggested that the regulation of *MAPK*, *AKT*, and *EGF/EGFR* may be potential underlying mechanisms. Furthermore, when an extra application of APP is given to the FASN-repressed CSCs, it leads to the restoration of their malignant characteristics. Collectively, APP serves as a novel finding for FASN downstream targets, promoting the advancement of CSCs. Targeting the FASN/APP axis could be beneficial in the development of anticancer medicines.

Sorafenib and lenvatinib have long served as the mainstay of HCC treatment (89), with regorafenib (90), ramucirumab (91), cabozantinib (90), and immune checkpoint inhibitors emerging as second-line treatments (92). Moreover, combination therapies have been extensively investigated and have shown promise in creating additional therapeutic options for patients (6). To seek alternative combined therapies that could improve the survival of liver cancer patients through targeting CSCs, we combined a conventional targeted therapy drug, sorafenib, with the FASN inhibitor, cerulenin, for potential treatment. It revealed that CSCs exhibited greater resistance than parental cells to sorafenib treatment, while the reverse effect was observed with cerulenin treatment. The synergistic effect of sorafenib and cerulenin was more pronounced in CSCs compared to the parental cells, and the use of combination treatments in clinical settings warrants additional investigation.

In conclusion, we found elevated markers of FA metabolism in liver CSCs and presented them as potential targets. Cerulenin attenuated stemness, tumorigenicity, migration, and invasion in CSCs and reprogrammed their metabolism. Further analyses indicated that FASN and APP were promising targets for anti-cancer therapy. Furthermore, we explored the possibility of combination therapy with the conventional targeted drug, sorafenib, and the metabolic inhibitor, cerulenin. The study demonstrated a greater synergistic effect in CSCs, which could be beneficial for developing novel therapeutic strategies.

## Data availability

All data generated or analyzed during this study are included in this published article.

## Supplemental Data

This article contains supplemental data

## Conflict of interest

The authors declare that they have no conflicts of interest with the contents of this article.

## Supplementary data

Supplemental FigureS1–S4
