# Supplementary figures and images for: Fatty acid synthase inhibitor cerulenin hinders liver cancer stem cell properties through FASN/APP axis as novel therapeutic strategies
Source: J Lipid Res. 2024 Sep 26;65(11):100660. doi: 10.1016/j.jlr.2024.100660 (PMC11539133; doi:10.1016/j.jlr.2024.100660)

Figure S1

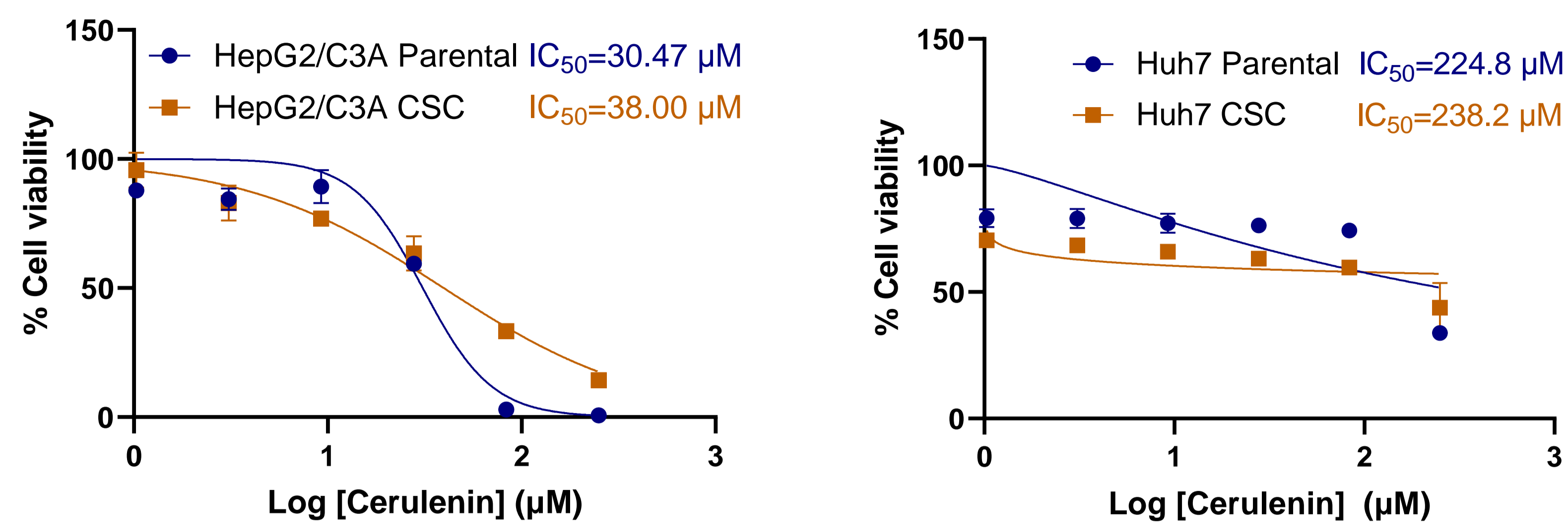

Figure S2

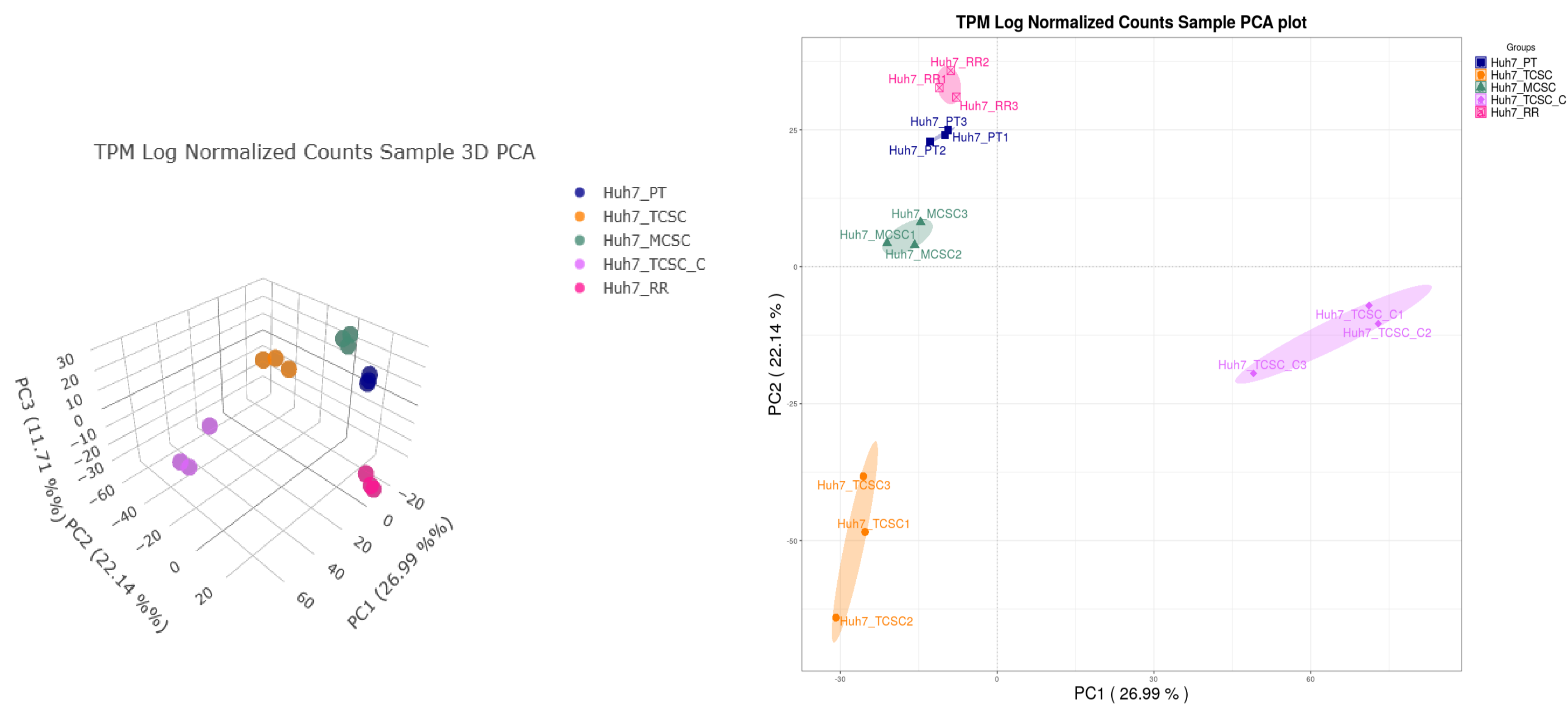

Figure S3

A

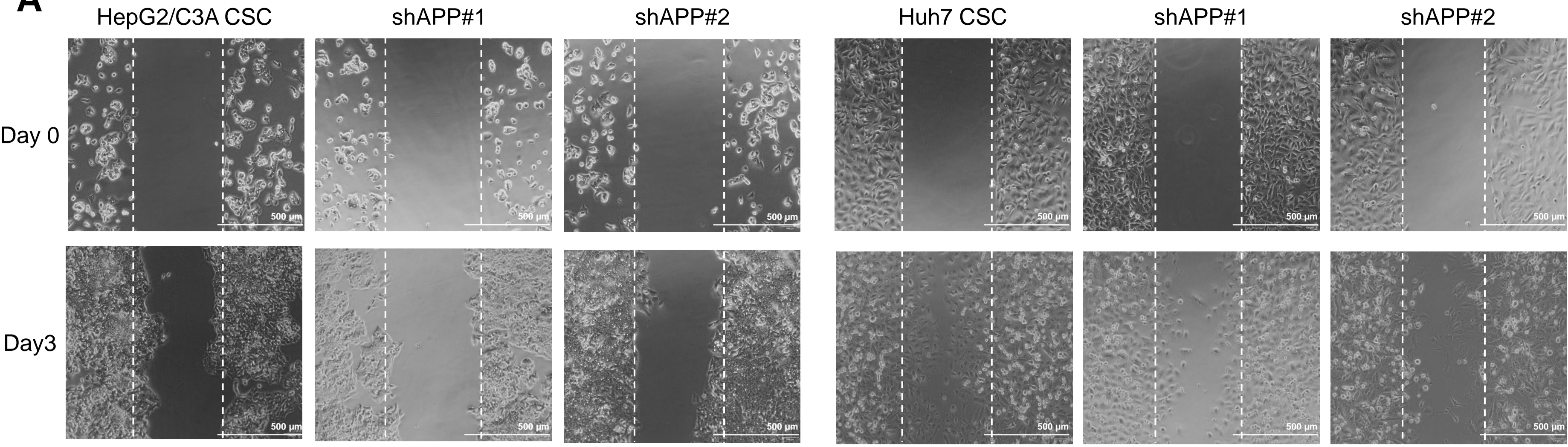

B

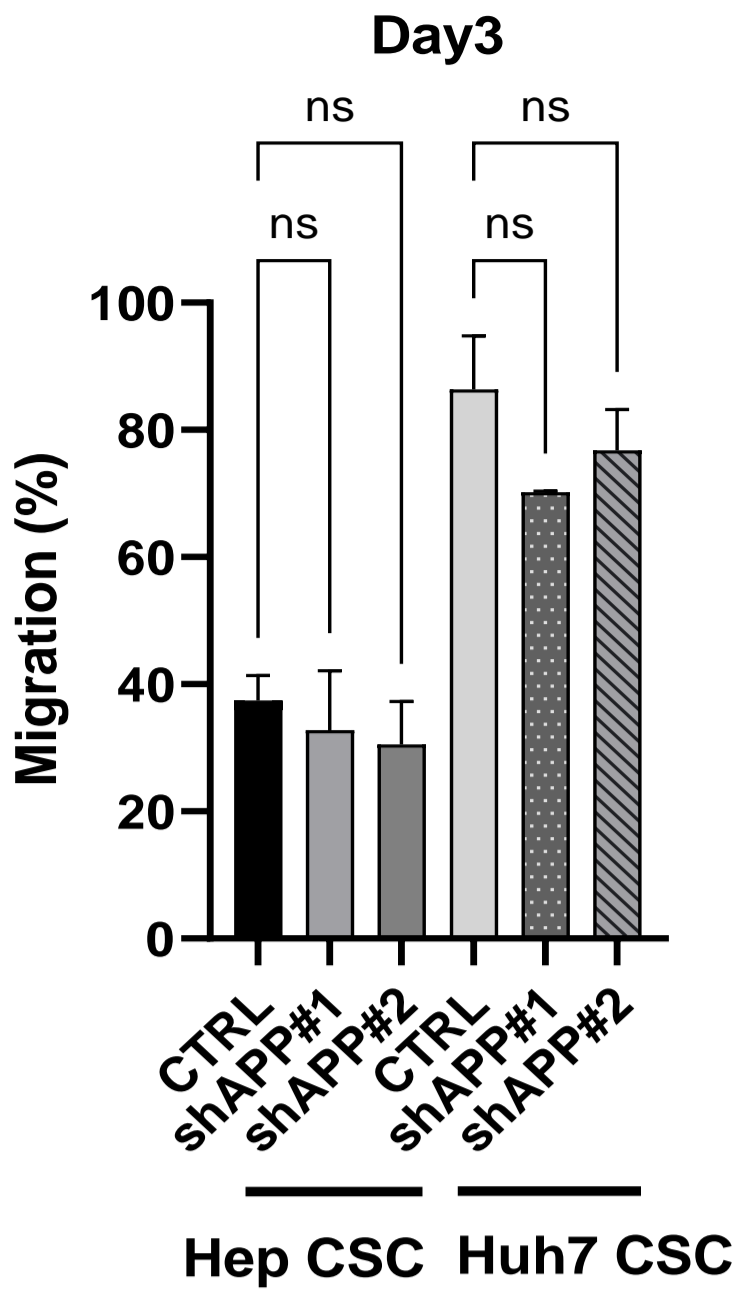

Figure S4

A

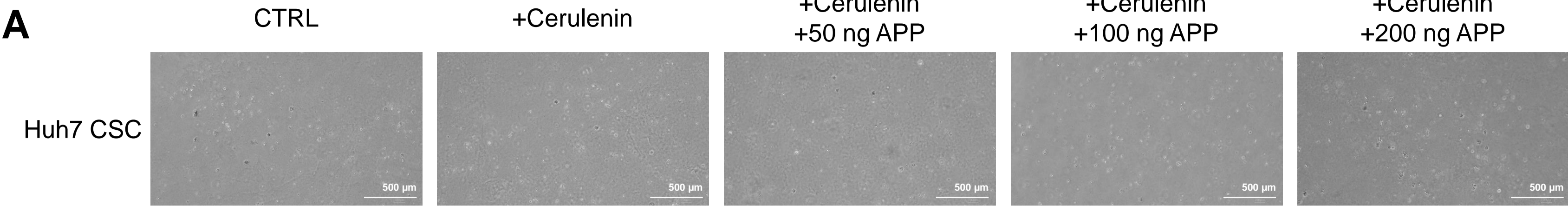

B

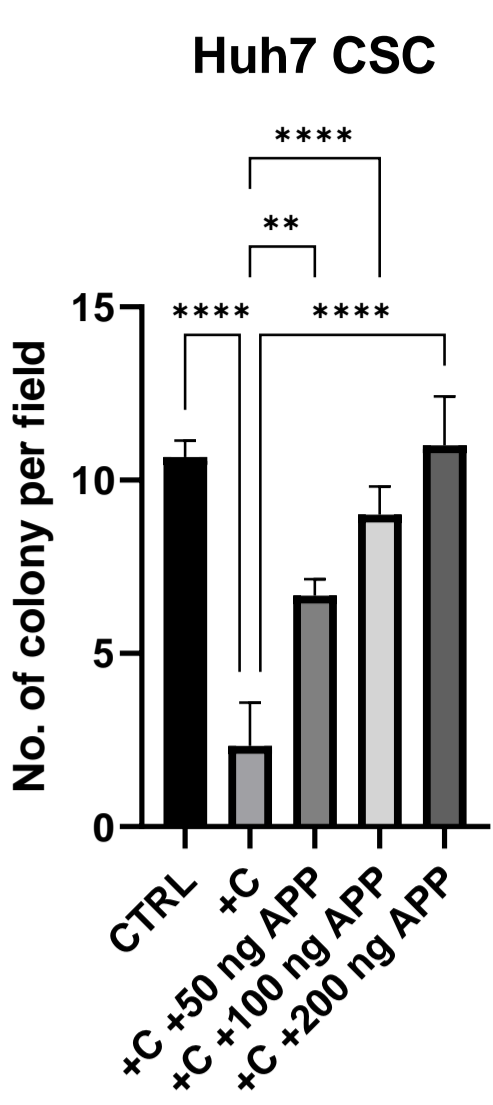

Supplement: Supplemental FigureS1–S4 [file mmc1.pdf]
